# Supplementary material for: A mixed methods study on men’s and women’s tuberculosis care journeys in Lusaka, Zambia—Implications for gender-tailored tuberculosis health promotion and case finding strategies
Source: PLOS Glob Public Health. 2023 Jun 16;3(6):e0001372. doi: 10.1371/journal.pgph.0001372 (PMC10275452; doi:10.1371/journal.pgph.0001372)
Supplement: S1 Table — (DOCX) [file pgph.0001372.s002.docx]

**S1 Table. Factors related to health-seeking and care engagement among men and women with newly diagnosed tuberculosis in Lusaka, Zambia.** Values represent adjusted predicted probabilities and associated 95% confidence intervals.

|  | **Overall**  **(95%CI)** | **Male**  **(95%CI)** | **Female**  **(95%CI)** | **Gender-specific difference***  **(95%CI)** |
| --- | --- | --- | --- | --- |
| **Did you contemplate seeking care sooner than you did?** |  |  |  |  |
| Yes | 53.9 (49.5, 58.3) | 53.6 (48.0, 59.1) | 54.6 (45.9, 63.4) | -1.1 (-11.9, 9.8) |
| **If you delayed seeking care, why?** |  |  |  |  |
| Thought symptoms were not serious and would get better on their own | 91.1 (87.6, 94.6) | 94.8 (91.5, 98.1) | 78.7 (65.0, 92.4) | **16.1 (1.4, 30.7)** |
| Thought symptoms were due to other causes (pollution, weather) | 89.7 (85.8, 93.6) | 88.2 (82.9, 93.4) | 92.6 (86.6, 98.6) | -4.4 (12.9, 4.0) |
| I did not know the symptoms of TB | 79.4 (74.4, 84.5) | 74.4 (67.5, 81.4) | 89.5 (82.2, 96.8) | **-15.1 (-26.0, -4.2)** |
| Preferred to try self-medication or home remedies first | 52.3 (46.3, 58.4) | 53.0 (45.8, 60.2) | 50.2 (36.1, 64.3) | 2.8 (-14.0, 19.6) |
| Lack of time | 48.1 (41.9, 54.4) | 50.7 (43.1, 58.3) | 40.9 (27.2, 54.5) | 9.8 (-6.7, 26.4) |
| It shows weakness to get help | 23.4 (17.9. 28.8) | 24.0 (-,-) | 21.8 (-.-) | 2.2 (-11.7, 16.0) |
| I had no one to assist me | 18.7 (13.7, 23.6) | 17.0 (11.4, 22.7) | 24.1 (11.1, 37.2) | -7.1 (-22.1, 8.0) |
| Thought symptoms were due to witchcraft, curse or fate | 15.0 (10.4, 19.5) | 16.2 (10.6, 21.8) | 10.7 (1.2, 20.2) | 5.5 (-6.3, 17.3) |
| Fear, embarrassment, or discrimination | 15.0 (10.2, 19.7) | 16.7 (10.4, 23.1) | 10.7 (2.1, 19.2) | 6.0 (-5.5, 17.6) |
| Previous bad experience with healthcare | 14.0 (9.7, 18.4) | 9.9 (5.3, 14.6) | 26.4 (12.5, 40.2) | **-16.4 (-31.8, -1.1)** |
| Worried it may be HIV or may be forced to test for HIV | 12.6 (8.4, 16.9) | 12.2 (7.4, 17.0) | 14.6 (1.5, 27.7) | 2.4 (-12.4, 17.1) |
| Too expensive | 5.1 (2.4, 7.9) | 2.6 (0, 5.3) | 11.4 (1.1, 21.7) | -8.7 (-19.9, 2.4) |
| **What were the reasons you first presented for help?** |  |  |  |  |
| I wanted to stay strong and healthy | 96.7 (95.1, 98.3) | 96.7 (94.6, 99.0) | 96.6 (93.2, 100) | 0.2 (-4.1, 4.6) |
| I was worried it could be something serious | 91.7 (89.0, 94.3) | 93.7 (90.6, 96.7) | 88.1 (82.2, 94.0) | 5.5 (-1.5, 12.5) |
| Loved ones/ friends were worried/ encouraged me to come | 84.9 (81.5, 88.3) | 82.3 (77.5, 87.1) | 89.4 (84.2, 94.6) | -7.1 (-14.6, 0.3) |
| I was weak/debilitated | 69.1 (64.7,73.3) | 66.8 (58.3,75.3) | 70.1 (64.6, 75.6) | -3.3 (-7.5, 1.4) |
| I could no longer work | 68.3 (63.9, 72.7) | 69.6 (64.1, 75.1) | 65.3 (56.1, 74.2) | 4.3 (-6.7, 15.4) |
| To set a good example for my loved ones | 58.9 (54.5, 63.3) | 57.5 (52.0, 62.9) | 62.8 (53.6, 72.0) | 5.3 (-5.9, 16.6) |
| I was worried it could be tuberculosis | 44.8 (40.2, 49.4) | 44.1 (38.4, 49.8) | 46.5 (37.4, 55.5) | 2.3 (-8.9, 13.6) |
| **Why did you choose the first facility?** |  |  |  |  |
| Close to home | 59.4 (55.0, 63.9) | 56.6 (50.9, 62.2) | 65.4 (57.3, 73.5) | -8.9 (-19.3, 1.5) |
| Good quality service | 49.9 (45.7, 54.1) | 50.7 (45.5, 55.8) | 48.0 (39.8, 56.2) | 2.7 (-7.5, 12.8) |
| Privacy/confidentiality | 41.3 (37.3, 45.3) | 42.4 (37.3, 47.5) | 39.1 (31.7, 46.6) | 3.3 (-6.2, 12.8) |
| Services are inexpensive | 39.3 (34.8, 43.7) | 40.7 (35.1, 46.4) | 36.3 (28.0, 44.6) | 4.4 (-6.1, 15.0) |
| Providers are nice/polite | 38.0 (33.8, 42.3) | 42.0 (36.6, 47.4) | 30.3 (23.1, 37.4) | **11.7 (2.4, 21.1)** |
| My friends and colleagues go there | 36.5 (31.9. 41.1) | 35.7 (29.9, 41.5) | 38.4 (29.1, 47.7) | -2.7 (-14.3, 8.8) |
| Familiar with the provider | 31.2 (26.7, 35.7) | 31.2 (25.5, 36.9) | 31.3 (22.5, 40.1) | -0.1 (-11.2, 10.9) |
| Short wait times | 30.5 (26.0, 34.9) | 31.9 (26.2, 37.6) | 27.5 (19.3, 35.7) | 4.4 (-6.2, 14.9) |
| Close to work | 29.0 (24.7, 33.2) | 30.8 (25.5, 36.1) | 24.0 (15.4, 32.6) | 6.8 (-3.9, 17.4) |

*Positive values indicate a higher probability among men, while negative values indicate a higher probability among women; values in bold indicate 95% confidence interval not overlapping zero, suggesting a significant difference at the level of p=0.05.

^”^(-,-)” indicates that the model failed to converge
