# Supplementary material for: A mixed methods study on men’s and women’s tuberculosis care journeys in Lusaka, Zambia—Implications for gender-tailored tuberculosis health promotion and case finding strategies
Source: PLOS Glob Public Health. 2023 Jun 16;3(6):e0001372. doi: 10.1371/journal.pgph.0001372 (PMC10275452; doi:10.1371/journal.pgph.0001372)
Supplement: S2 Table — (DOCX) [file pgph.0001372.s003.docx]

**S2 Table. Self-reported TB symptoms among men and women with newly diagnosed tuberculosis in Lusaka, Zambia.** Values represent adjusted predicted probabilities and associated 95% confidence intervals.

|  | **Overall**  **(95%CI)** | **Male**  **(95%CI)** | **Female**  **(95%CI)** | **Gender-specific difference***  **(95%CI)** |
| --- | --- | --- | --- | --- |
| **Cough** |  |  |  |  |
| ≥2 weeks | 80.0 (76.2, 83.8) | 77.8 (72.4, 83.3) | 83.2 (77.1, 89.2) | -5.3 (-14.0, 3.3) |
| <2 weeks | 11.0 (8.0, 14.0) | 12.0 (7.9, 16.2) | 9.3 (4.5, 14.1) | 2.7 (-4.1, 9.5) |
| None | 9.0 (6.3, 11.7) | 10.2 (6.1, 14.3) | 7.5 (3.6, 11.5) | 2.6 (-3.4, 8.7) |
| **Fevers** | 83.0 (79.5, 86.5) | 83.3 (78.5, 88.0) | 82.6 (76.5, 88.7( | 0.6 (-7.7, 8.9) |
| **Night sweats** | 89.3 (86.4, 92.1) | 93.8 (90.7, 96.9) | 80.9 (72.8, 89.1) | **12.8 (3.6, 22.1)** |
| **Weight loss** | 96.7 (95.0, 98,5) | 97.1 (94.8, 99.3) | 96.3 (93.1, 99.5) | 0.8 (-3.5, 5.0) |
| **Chest pain** | 73.5 (69.4, 77.6) | 77.0 (71.6, 82.3) | 67.3 (58.7, 75.9) | 9.7 (-1.3, 20.6) |
| **Shortness of breath** | 78.8 (74.8, 82.7) | 77.5 (72.2, 82,9) | 80.9 (74.0, 87.7) | -3.3 (-12.7, 6.0) |
| **Sputum production** | 68.5 (64.2, 72.8) | 67.5 (61.8, 73.2) | 70.4 (62.5, 78.2) | -2.9 (-13.3, 7.5) |
| **Fatigue** | 82.8 (79.1, 86.4) | 83.2 (78.4, 88.1) | 81.9 (74.9, 89.0) | 1.2 (-7.9, 10.5) |
| **Loss of appetite** | 92.3 (89.7, 94.8) | 92.6 (89.4, 95.7) | 91.3 (85.0, 97.5) | 1.3 (-6.1, 8.8) |
| **Body pain** | 52.5 (47.9, 57.1) | 59.2 (50.5, 67.8) | 49.3 (43.4, 55.1) | 9.9 (-1.3, 21.1) |
| **Number of TB symptoms^#^** |  |  |  |  |
| 1-2 | 1.3 (0.2, 2.3) | 0.5 (-0.4, 1.3) | 2.2 (0, 4.3) | -1.7 (-4.1, 0.7) |
| 3 | 6.8 (4.4, 9.1) | 7.1 (3.6, 10.5) | 6.4 (-5.8, 18.5) | 0.7 (-4.8, 6.2) |
| ≥4 | 92.0 (89.4, 94.6) | 92.5 (89.0, 96.1) | 91.6 (59.4, 100) | 1.1 (-4.9, 6.9) |

*Positive values indicate a higher probability among men, while negative values indicate a higher probability among women; values in bold indicate 95% confidence interval not overlapping zero, suggesting a significant difference at the level of p=0.05.
^#^Number of the following symptoms: cough, chest pain, shortness of breath, fevers, weight loss, night sweats
