# Supplementary material for: A mixed methods study on men’s and women’s tuberculosis care journeys in Lusaka, Zambia—Implications for gender-tailored tuberculosis health promotion and case finding strategies
Source: PLOS Glob Public Health. 2023 Jun 16;3(6):e0001372. doi: 10.1371/journal.pgph.0001372 (PMC10275452; doi:10.1371/journal.pgph.0001372)
Supplement: S3 Table — (DOCX) [file pgph.0001372.s004.docx]

**S3 Table. Measures of satisfaction with tuberculosis services among men and women with newly diagnosed tuberculosis in Lusaka, Zambia.** Values represent adjusted predicted probabilities and associated 95% confidence intervals.

|  | **Overall**  **(95%CI)** | **Male**  **(95%CI)** | **Female**  **(95%CI)** | **Gender-specific difference***  **(95%CI)** |
| --- | --- | --- | --- | --- |
| **How satisfied are you with the services you received?** |  |  |  |  |
| Satisfied | 95.2 (-.-) | 95.1 (-.-) | 95.4 (-.-) | -0.2 (-5.2, 4.8) |
| Not satisfied | 4.0 (-.-) | 4.1 (-.-) | 4.0 (-.-) | 0.1 (-4.4, 4.6) |
| Unsure | 0.8 (-.-) | 0.8 (-.-) | 0.7 (-.-) | 0.1 (-2.0, 2.3) |
| **Does this facility meet your gender-specific needs?** |  |  |  |  |
| Yes | 91.7 (-.-) | 92.9 (-.-) | 88.9 (-.-) | 4.0 (-3.4, 11.4) |
| No | 3.5 (-.-) | 3.9 (-.-) | 2.2 (-.-) | 1.7 (2.3, 5.6) |
| Unsure | 4.8 (-.-) | 3.2 (-.-) | 8.9 (-.-) | -5.7 (-12.2, 0.8) |
| **Would you recommend this facility to a friend?** |  |  |  |  |
| Yes | 92.4 (-.-) | 91.0 (-.-) | 96.1 (-.-) | -5.1 (-10.0, -1.2) |
| No | 3.3 (-.-) | 3.9 (-.-) | 1.5 (-.-) | 2.5 (-0.6, 5.5) |
| Unsure | 4.3 (-.-) | 5.1 (-.-) | 2.5 (-.-) | 2.6 (-1.5, 6.7) |

*Positive values indicate a higher probability among men, while negative values indicate a higher probability among women; values in bold indicate 95% confidence interval not overlapping zero, suggesting a significant difference at the level of p=0.05. ^”^(-,-)” indicates that the model failed to converge.
