# Supplementary material for: A mixed methods study on men’s and women’s tuberculosis care journeys in Lusaka, Zambia—Implications for gender-tailored tuberculosis health promotion and case finding strategies
Source: PLOS Glob Public Health. 2023 Jun 16;3(6):e0001372. doi: 10.1371/journal.pgph.0001372 (PMC10275452; doi:10.1371/journal.pgph.0001372)
Supplement: S4 Table — (DOCX) [file pgph.0001372.s005.docx]

**S4 Table. Preferred communication channels and persons for health-related information and decision making among men and women with newly diagnosed tuberculosis in Lusaka, Zambia.** Values represent adjusted predicted probabilities and associated 95% confidence intervals.

|  | **Overall**  **(95%CI)** | **Male**  **(95%CI)** | **Female**  **(95%CI)** | **Gender-specific difference***  **(95%CI)** |
| --- | --- | --- | --- | --- |
| **What are the best channels to reach you with health-related information?** |  |  |  |  |
| Plays in the community | 96.2 (94.4, 98.0) | 97.7 (95.9, 99.5) | 93.3 (88.8, 97.8) | 4.4 (-5.4, 9.4) |
| Brochures | 94.1 (80.8, 87.5) | 84.7 (80.2, 89.2) | 83.2 (77.1, 89.3) | 1.5 (-6.6, 9.6) |
| Radio | 86.1 (83.1, 89.2) | 86.4 (82.3, 90.6) | 85.7 (80.1, 91.2) | 0.8 (-6.7, 8.2) |
| TV | 84.4 (81.3, 87.5) | 81.6 (77.2, 86.1) | 88.6 (83.9, 93.3) | **-7.0 (-13.8, -0.1)** |
| Billboards | 79.1 (75.5, 82.7) | 80.3 (75.7, 84.9) | 76.9 (70.1, 83.7) | 3.5 (-5.2, 12.1) |
| Social media | 72.3 (68.3, 76.3) | 74.2 (69.2, 79.2) | 68.1 (59.7, 76.6) | 6.0 (-4.3, 16.4) |
| Newspapers/magazines | 62.0 (57.4, 66.4) | 63.9 (58.2, 69.5) | 57.8 (48.7, 66.9) | 6.1 (-5.2, 17.4) |
| **Person(s) who influence health decisions** |  |  |  |  |
| Nobody (only myself) | 32.7 (28.8. 36.6) | 37.9 (32.9, 43.0) | 23.8 (32.9, 43.0) | 14.1 (5.8, 22.4) |
| **If someone influences your health decisions, who?** |  |  |  |  |
| Other family members | 93.3 (90.5, 96.0) | 94.2 (91.1, 97.3) | 89.7 (81.0, 98.3) | 4.5 (-5.0, 14.1) |
| Friends | 71.9 (66.8, 77.1) | 73.0 (66.7, 79.3) | 69.1 (57.7, 80.5) | 3.9 (-10.0, 17.8) |
| Healthcare workers | 63.7 (58.8, 68.5) | 57.7 (46.7, 68.6) | 65.6 (59.9, 71.4) | -8.0 (-21.0, 5.1) |
| Spouse/partner | 57.3 (53.3, 61.3) | 59.8 (54.7, 64.9) | 51.5 (43.2, 59.9) | 8.3 (-2.3, 18.9) |
| Religious leaders | 52.4 (46.8. 58.1) | 47.8 (40.4, 55.2) | 62.9 (51.6, 74.9) | **-15.1 (-29.8. -0.4)** |
| Coworkers | 47.2 (41.4, 52.9) | 54.4 (46.8, 61.9) | 31.9 (21.1, 42.7) | **22.4 (8.4, 36.5)** |
| Neighbors | 45.7 (40.0, 51.4) | 50.4 (43.0, 57.8) | 35.7 (24.7, 46.6) | **14.7 (0.5, 28.9)** |

*Positive values indicate a higher probability among men, while negative values indicate a higher probability among women; values in bold indicate 95% confidence interval not overlapping zero, suggesting a significant difference at the level of p=0.05. ^”^(-,-)” indicates that the model failed to converge.
