# Supplementary material for: A mixed methods study on men’s and women’s tuberculosis care journeys in Lusaka, Zambia—Implications for gender-tailored tuberculosis health promotion and case finding strategies
Source: PLOS Glob Public Health. 2023 Jun 16;3(6):e0001372. doi: 10.1371/journal.pgph.0001372 (PMC10275452; doi:10.1371/journal.pgph.0001372)
Supplement: S2 Appendix — (DOCX) [file pgph.0001372.s007.docx]

**S2 Appendix. In-depth interview guide for newly diagnosed tuberculosis patients**

**Introduction**

Good morning/afternoon. Thank you for taking the time to participate in this interview. My name is ______.

**Purpose of this interview.**

Before we begin, I want to tell you a little bit about today’s interview and what we are going to do here today. We are gathering information from current TB patients to understand the challenges that individuals face in receiving a timely TB diagnosis when they have symptoms that could be TB. The study also aims to better understand the experiences and preferences of current TB patients like yourself. The information from today’s interview will help us to develop ways to improve care for TB patients and also improve TB diagnosis and control in the community.

We anticipate that the interview will last approximately 45-60 minutes. You will be given 100ZMW upon completion of the interview. We will be recording the interview so that we can go back and review what was said later. We will be taking notes to help us in case the recording fails.

Do you have any questions so far? If not, then I would like to begin.

|  | **Questions** | **Probes** | **Rationale** |
| --- | --- | --- | --- |
| **Introduction** | Tell me a little bit about your home life. | 1a. Where is your home?  1b. Who all live there with you?  1.c. How do you spend your time? | Background |
| **Pre-Diagnosis TB Knowledge/ Attitudes** | 1. Before you were diagnosed, what did you know about TB? 2. Did you know anyone who had TB? 3. What were your thoughts about TB before you were diagnosed? | 2a. What had you heard? From whom?  3a. Did this influence what you thought about TB?  3b. Did this influence your decision to get help for your illness?  4a. What do people in your community say about TB? | Beliefs about TB and TB treatment  Social opportunity  Impact of TB Stigma |
| **Pre-Diagnosis Health and Relationship to Health Care** | Before you were ill from TB, how was your health? | 5a. If good, what do you think contributed to this good health? If poor, why?  5b. Did you ever see a doctor for any reason?  5c. Other than a doctor, who else offered you advice on your health? | Health/health care seeking behaviors prior to TB will impact relationship to TB care |
| **TB Illness Experience** | 1. How did your illness begin? | 6a. What were your symptoms?  6b. What did you think was going on? |  |
| **Decision-making around accessing care for TB** | 1. Tell me about your experience with seeking care for your symptoms. What made you think you might need help?   *[keep asking, and then what happened?]* | 7a. How soon after your symptoms started did you seek care? Why did you wait?  7b. [keep asking] Where did you go? Why did you go there? What did they do for you?  7c. If current care location not the first place participant sought help: Why did you ultimately come here? | TB pathways |
| **Diagnosis Experience** | 1. Can you walk me through what happened on the day you were diagnosed and the day the results were shared with you? | 8a. What tests were conducted? Did you know what was happening? Did you have to come back for your result? How difficult was that for you?  8b. Who gave you your result? What did they say to you? What was your reaction?  8c. Did you have concerns? Did you discuss your concerns? | What messaging did patient hear from health care providers? |
| **Post-Diagnosis and Treatment Experience** | 1. What kinds of thoughts and feelings did you have in the days after you were diagnosed about having TB and taking treatment? 2. What was your experience like starting treatment? 3. What was your experience like at the clinic/hospital? 4. For HIV positive: How did being HIV-positive affect your ability to seek care for TB? 5. How has having TB changed your daily activities? | 9a. Who did you tell? What were their reactions?  9b. Was there anyone you did not tell on purpose? Why?  9c. Did you feel supported? Who gave you support?  10a. How long after being diagnosed did you start? If more than a few days, what contributed to this delay?  11a. How did clinic staff treat you? How could the clinic/hospital experience have been better?  13a. Has it affected your ability to make money/care for your family? | Any TB stigma? |
| **Reflections** | 1. Looking back, what about TB or its treatment would it have been helpful for you to know sooner? 2. What do you think made it hard to seek care? Did anything make it easier? 3. Some **men (if male)** or women **(if female)** have told me that they think other **men/women** might wait to get help for an illness like TB. What do you think about that? Why might that be? 4. Is there anything you would suggest to improve the process of diagnosis and starting TB treatment? 5. Some people have suggested the following strategies as ways to get patients into TB care sooner? What do you think about that? 6. Thinking about your experience with TB, what would you want friends or loved ones to know? | 14a. What would have been the best ways to reach you with information? (TV, radio, newspaper, social media?)  16a. Is there anything specifically about being a **man (if male)** or women **(if female)** makes it difficult to get help for an illness?  16b. Do you think that being viewed as weak or vulnerable plays a role?  16c. Do you think the pressure to generate money for oneself and/or your family plays a role?  18a. A mobile clinic/stand in the community for rapid TB testing (market, minibus stand).  18b. Offering a small amount of money to get tested for TB.  18c. Training current TB patients and providing them a small amount of money to reach out to their peers that may have TB for testing?  19a. What about health care providers? | Responsibilities  Gender expectations |
| **Wrap Up** | 1. We have talked about many things related to TB and TB care? Are there any final thoughts you would like to share with me? Do you have any questions? |  |  |
