# Supplementary material for: A mixed methods study on men’s and women’s tuberculosis care journeys in Lusaka, Zambia—Implications for gender-tailored tuberculosis health promotion and case finding strategies
Source: PLOS Glob Public Health. 2023 Jun 16;3(6):e0001372. doi: 10.1371/journal.pgph.0001372 (PMC10275452; doi:10.1371/journal.pgph.0001372)
Supplement: S3 Appendix — (DOCX) [file pgph.0001372.s008.docx]

**S3 Appendix. Codebook for qualitative data analysis**

| **Code** | **Description** |
| --- | --- |
| **Pre-diagnosis and health-seeking** | |
| Health attitudes and experiences before TB | Apply this code when participants discuss how their health was prior to their TB illness as well as when they discuss any healthcare experiences (positive or negative) they had prior to their TB illness. |
| Pre-diagnosis knowledge, attitudes, beliefs and misconceptions towards TB   - Sources of information | Apply this code when participants discuss their knowledge, attitudes, beliefs and misconceptions prior to being diagnosed with TB (including their perceived risk of getting TB, symptoms, how it’s transmitted, treatment, etc). Also, apply this code when participants mention attitudes, beliefs and misconceptions that they have seen others in their community hold. Include discussions of where they themselves or members of the community tend to receive/learn TB-specific knowledge and education.   - Apply this subcode when participants discuss where they themselves or members of the community tend to receive/learn TB-specific knowledge and education. |
| TB symptoms | Apply this code when participants discuss how they experienced their TB illness, including any symptoms they experienced as part of their illness. |
| Barriers and delays to care seeking | Apply this code whenever a participant mentions any factor or reasons (barriers) that relate to/ contribute to delays in health seeking for their TB-related illness (e.g., the time between symptom onset and initial care seeking to any type of facility or provider). |
| Care seeking decision making | Apply this code when participants note the reasons that motivated them to seek care for their TB illness when they did. Also apply it when participants note what they think motivates persons in their community to seek care for TB when they do. Do not apply this code to what factors keep/kept participants from seeking care sooner, instead see code ‘Barriers and delays to care seeking.’ |
| Reasons for choosing facility | Apply this code when participants discuss the reasons for choosing a given facility/provider for evaluating or treating their TB illness. Also apply this code when participants discuss the specific reasons why or how they came to present to one of the study-related health facilities. Also apply this code when participants discuss what tends (reasons, conditions, etc) to bring community members to that specific facility. |
| **Diagnosis experience** | |
| Experiences with care seeking and diagnosis | Apply this code when participants discuss their personal experience of what it was like to be evaluated for and diagnosed with TB. This may include tests, investigations, empiric therapy, service characteristics (e.g., wait times, friendliness of providers, etc). |
| Barriers and delays to diagnosis | Apply this code whenever a participant mentions any factor or reasons (barriers) that relate to/ contribute to delays in TB diagnosis (e.g., the time after an individual has first sought care for their illness, but before their TB is diagnoses). |
| Reaction to TB diagnosis | Apply this when participants discuss how they felt, reacted or what concerns they had upon learning their TB diagnosis. |
| **Post-diagnosis and treatment experience** | |
| Barriers and delays to treatment | Apply this code whenever a participant mentions any factor or reasons (barriers) that relate to/ contribute to delays in TB treatment initiation (e.g., the time after an individual’s TB is diagnosed but before they start TB treatment). |
| Disclosure | Apply this code when participants discuss either to whom they disclosed (and what that that process was like) or concerns related to disclosing to one or more persons (and associated reasons why). |
| TB medication experience   - Adherence challenges | Discuss this code when participants discuss their own experiences including adherence challenges related to TB treatment (either the medication itself or the process of accessing medications and related appointments), or when participants describe experiences related to TB treatment that they have observed or have been told about.   - Apply this sub-code when participants mention any challenges related to consistently taking and completing their TB therapy. |
| **Across the TB care pathway** | |
| Alcohol use, bars and smoking | Apply this code when smoking, alcohol use or bar attendance is mentioned in any context by any participant. |
| COVID-19 | Apply this code when a participant mentions anything in relation to COVID-19. Code appropriately if discussion highlights other parts of interest. |
| Curses or fate | Apply this code when a participant makes any mention to TB being related to curses, witchcraft or fate (e.g., that TB is predetermined and/or in some way destined). |
| Depression | Apply this code when a participant mentions feeling sad, low or depressed in any context related to TB (pre-health seeking, at time of diagnosis, during treatment, etc). |
| HIV | Apply this code when HIV is mentioned by any participant in any context. This may include how being HIV positive may affect any aspect of the TB care journey, or how HIV-related stigma may affect attitudes about TB, including healthcare seeking for TB. |
| Impact of TB on daily life | Apply this code when participants discuss how TB has affected aspects of their daily life or concerns around how TB could affect aspects of one’s daily life. This includes impacts on social interactions, work/employment and income as well as impacts on food security, housing security, etc. |
| Misfits | Apply this code when a participant statement is of interest or possible importance but does not fit any existing codes. |
| Quotes | Apply this to outstanding, notable, sound-byte worthy quotes. Code appropriately if discussion highlights other parts of interest. |
| Social influences, support and opportunity   - Family/friend with TB | Apply this code when participants mention the presence or absence of social support (friends, colleagues, loved ones), including persons who serve as role models and/or encouraged and supported health seeking, diagnosis, or treatment. Also apply this code when participants note how friends, colleagues, loved ones, specifically influence their knowledge and/or health-related behaviors.   - Apply this subcode when participants mention that they have a family member and/or friend who has had TB and any statements related to how the influenced their knowledge of TB. |
| Stigma | Apply this code when participants note in any context, stigma associated with, anticipated, or experienced related to TB. This includes individuals’ beliefs and experiences as well as community beliefs/perceptions. Also apply this code to any statements that discuss HIV-related stigma in the context of TB (in addition to coding using the HIV parent code above). |
| **Gendered differences** |  |
| Men’s knowledge, health and behaviors | Apply this code whenever any participant mentions any differences between men and women in any way focusing on men (e.g., knowledge, attitudes, behaviors). Also apply when participants specifically note that some aspect does NOT differ between men and women. |
| Male-specific engagement strategies | Apply this code whenever any participant mentions specific strategies to better reach/engage men (at any step in the TB care pathway). |
| Female’s knowledge, health and behaviors | Apply this code whenever any participant mentions any differences between men and women in any way focusing on women (e.g., knowledge, attitudes, behaviors). Also apply when participants specifically note that some aspect does NOT differ between women and men. |
| Female-specific engagement strategies | Apply this code whenever any participant mentions specific strategies to better reach/engage women (at any step in the TB care pathway). |
| **Recommendations and suggestions** |  |
| Advice related to TB | Apply this code when any participant shares advice related to TB, including knowledge, recommendations, or reflects on lessons learned from their own experiences. |
| Reaching community members | Apply this code a participant describes different messages and methods to best reach community members with TB and other health-related messages. Also apply this code when participants mention what has been effective for reaching community members in the past. |
| Suggestions for improving TB testing   - Best strategy | Apply this code when participants provide a recommendation for how TB diagnosis can be improved to either reach and detect TB in more individuals and/or identify TB sooner and ways in which TB treatment could be made easier/improved for themselves of other persons with TB. This may include recommendations that apply to community-based approaches, or recommendations that apply to existing health facility services and facility-based approaches.   - Apply this subcode in reference to the rank order exercise (e.g., which of these strategies do you think is best?) |
| Suggestions for improving TB treatment | Apply this when participants describe ways in which TB treatment could be made easier/improved for themselves of other persons with TB. This may include aspects related to the treatment itself (less pills, shortened duration), access to treatment (decentralization etc), as well as adjunctive measures (financial or food support, etc). |
